# A Cross-Tissue Transcriptome-Wide Association Study Identifies Novel Susceptibility Genes for Juvenile Idiopathic Arthritis in Asia and Europe

**DOI:** 10.3389/fimmu.2022.941398

**Published:** 2022-07-28

**Authors:** Jiawen Xu, Jun Ma, Yi Zeng, Haibo Si, Yuangang Wu, Shaoyun Zhang, Bin Shen

**Affiliations:** Orthopedic Research Institute, Department of Orthopedics, Sichuan University West China Hospital, Chengdu, China

**Keywords:** transcriptome-wide association study, juvenile idiopathic arthritis, mRNA expression profiles, gene ontology, pathway enrichment

## Abstract

**Background:**

Juvenile idiopathic arthritis (JIA) is the most common rheumatic disease in children, and its pathogenesis is still unclear. Genome-wide association studies (GWASs) of JIA have identified hundreds of risk factors, but few of them implicated specific biological mechanisms.

**Methods:**

A cross-tissue transcriptome-wide association study (TWAS) was performed with the functional summary-based imputation software (FUSION) tool based on GWAS summary datasets (898 JIA patients and 346,102 controls from BioBank Japan (BBJ)/FinnGen). The gene expression reference weights of skeletal muscle and the whole blood were obtained from the Genotype-Tissue Expression (GTExv8) project. JIA-related genes identified by TWAS findings genes were further compared with the differentially expressed genes (DEGs) identified by the mRNA expression profile of JIA from the Gene Expression Omnibus (GEO) database (accession number: GSE1402). Last, candidate genes were analyzed using functional enrichment and annotation analysis by Metascape to examine JIA-related gene sets.

**Results:**

The TWAS identified 535 significant genes with *P* < 0.05 and contains 350 for Asian and 195 for European (including 10 genes both expressed in Asian and European), such as *CDC16* (*P* = 1.72E-03) and *PSMD5-AS1* (*P* = 3.65E-02). Eight overlapping genes were identified based on TWAS results and DEGs of JIA patients, such as *SIRPB1* (*P*_TWAS_ = 4.21E-03, *P*_DEG_ = 1.50E-04) and *FRAT2* (*P*_TWAS_ = 2.82E-02, *P*_DEG_ = 1.43E-02). Pathway enrichment analysis of TWAS identified 183 pathways such as cytokine signaling in the immune system and cell adhesion molecules. By integrating the results of DEGs pathway and process enrichment analyses, 19 terms were identified such as positive regulation of T-cell activation.

**Conclusion:**

By conducting two populations TWAS, we identified a group of JIA-associated genes and pathways, which may provide novel clues to uncover the pathogenesis of JIA.

## Introduction

Juvenile idiopathic arthritis (JIA) is a group of arthritis of unknown origin that begins before the age of 16 and persists for more than 6 weeks (1). JIA is the most common childhood chronic rheumatic disease, has a prevalence of 3.8-400 cases per 100,000 in high-income countries, and causes damage to physical development, psychiatric development, and disabilities in children (2). The high prevalence and severe consequences of JIA bring enormous social and economic burdens to society, but there is still no clear underlying mechanism of JIA development.

The pathogenesis of JIA remains unclear, but it is thought to be multifactorial with complex interactions between genetic susceptibility and environmental factors (3). It has been shown that JIA is similar to other autoimmune diseases, with which it shares susceptibility genes, mainly in the human leukocyte antigen (HLA) region (4–6). In addition, evidence from twin and familial studies suggested a genetic predisposition for JIA, with a heredity of 13% (7, 8). In recent years, an increasing number of studies have focused on the genetic mechanism of JIA. A genome-wide linkage study of 121 JIA-affected sibling-pair families suggested that genes in the HLA influence the risk of JIA (9). In the era of genome-wide association studies (GWASs), this novel approach has identified several JIA-associated loci and genes, such as *VTCN1*, 3q13 within *C3orf1*, 10q21 near *JMJD1C*, 4q31 (10, 11). However, most of the variants at loci are often located in non-coding region (12).

Gene expression is a key step linking DNA sequence variation to phenotypes, which limits the use of GWASs in evaluating the risk of disease. Therefore, the specific biological mechanisms need to be further investigated. A previous study showed that many genetic variants play vital roles in complex traits by modulating gene expression (13). In 2018, a new omics analysis method called transcriptome-wide association studies (TWASs) emerged, which leverage expression reference panels (eQTL cohorts with expression and genotype data) to discover gene–trait associations in GWAS datasets, providing a powerful strategy that integrates GWASs and gene expression references to identify significant expression-trait associations (14–16). It could reduce large of proportion non-sense results obtained from GWAS and enhance the ability to discovery novel gene-related disease (17). Recently, TWASs have been successfully used by a growing number of researchers to identify genes associated with complex diseases and traits, such as osteoarthritis and rheumatoid arthritis (16–19).

JIA is a heterogeneous inflammatory rheumatic condition including seven different categories and has different and prevalence between population (20). To overcome the difference of heterogeneous condition and ethnicity, we utilized TWAS based on two large-scale JIA GWAS datasets from Asian and European populations in this study. To validate the TWAS results, the candidate genes identified by TWAS were further compared with the mRNA expression profiles of JIA. Finally, we re-evaluated the expression of the TWAS-identified genes and performed a functional examination. We hope our study will provide novel clues for the genetic mechanism of JIA between populations.

## Methods and Materials

### Genome-Wide Association Studies Summary Data of Juvenile Idiopathic Arthritis

Recent large-scale GWASs and meta-analysis in Asian (21) and European (22) populations of JIA were used here. Briefly, GWAS summary dataset of Asian contains 110 diagnosed JIA patients and 173,268 controls from the BioBank Japan (BBJ) (21). GWAS summary dataset of European was obtained from the FinnGen study, which was launched in Finland in 2017, including 788 diagnosed JIA and 172,834 controls of Finnish participants (22). All cases were defined by the code M13 in the International Classification of Diseases—Tenth Revision. Genotyping was conducted using commercial platforms, such as the Illumina HumanOmniExpressExome BeadChip, typed at 30,390,156 variants (including 13,530,797 for Asian and 16,859,359 for European) analyzed in total. Detailed information on the subjects, genotyping, imputation, and quality control can be found in the published studies (21, 22).

### Transcriptome-Wide Association Study Analysis of Juvenile Idiopathic Arthritis

Tissue-related TWAS analysis of JIA was carried out by using functional summary-based imputation software (FUSION) *via* integrating the GWAS summary datasets of JIA and pre-computed gene expression weights reference of skeletal muscle and whole blood. Using pre-computed gene expression weights and GWAS summary datasets, FUSION can evaluate the gene expression associations between each gene and target disease (23). Specifically, the gene expression weights of skeletal muscle and whole blood were calculated using the prediction models of FUSION. FUSION computed TWAS expression weights using five linear models, namely, best linear unbiased prediction (BLUP), Bayesian sparse linear mixed model (BSLMM), least absolute shrinkage and selection operator (LASSO), Elastic Net, and top single nucleotidepolymorphisms (SNPs) from the reference expression panels (i.e., GTExv8). When performing transcriptomic imputation, FUSION calculated an out-sample R2 using a fivefold cross-validation of each model to determine the best performing prediction model for a gene. Then, the calculated expression weights were combined with GWAS results to impute association statistics between gene expression levels and target diseases. The association testing statistics between predicted gene expression and target diseases was calculated as ZTWAS = w’Z/(w’Lw)1/2. “Z” denotes the scores of JIA, and “w” denotes the weights. “L” denotes the SNP-correlation linkage disequilibrium (LD) matrix (23). In the present study, a TWAS *P*-value was calculated for each gene within skeletal muscle, whole blood for Asian and European populations, respectively. The genes with *P* < 0.05 was considered as significant.

### mRNA Expression Profiles of Juvenile Idiopathic Arthritis

The differentially expressed genes (DEGs) were derived from genome-wide mRNA expression profiles of JIA. The JIA expression data were downloaded from the Gene Expression Omnibus (GEO) Datasets GSE1402 (https://www.ncbi.nlm.nih.gov/geo/query/acc.cgi?acc=GSE1402) and the corresponding reference (24). In brief, JIA samples were obtained from 35 JIA patients, including 10 pauciarticular JIA patients and 25 polyarticular JIA patients. Eleven control samples were obtained from healthy individuals. The mRNA samples were processed according to the Affymetrix GeneChip Expression Analysis Technical Manual (Affymetrix; Santa Clara, CA) (24). In this study, DEGs were analyzed by the GEO2R tool. GEO2R has a simple interface that allows users to perform sophisticated R-based analysis of GEO data to help identify and visualize differential gene expression (25). Genes were identified when the following two conditions were met: adjusted *P* < 0.05 by the moderated t statistic and |log2FC| > 1.

### Functional Enrichment and Annotation Analysis

Gene Ontology (GO) and pathway enrichment analysis of the genes identified by the TWAS analysis and mRNA expression profiles were performed by the Metascape tool (https://metascape.org/gp/index.html#/). Metascape had been designed to allow experimentalists to apply powerful computational analysis pipelines to analyze and interpret large-scale datasets, facilitating functional exploration that includes GO and pathway analysis (26). First, candidate genes identified by TWAS were analyzed by the Metascape tool. Second, to find the common GO terms and Kyoto Encyclopedia of Genes and Genomes (KEGG) pathways, we further compared the Metascape results of TWAS analysis and mRNA expression profiles. Enrichment analysis was based on Fisher’s exact test and the calculation of *P*-values. Terms with *P* < 0.05 were considered significant.

## Results

### Transcriptome-Wide Association Study Analysis Results of the Asian Population

**Figure 1:**
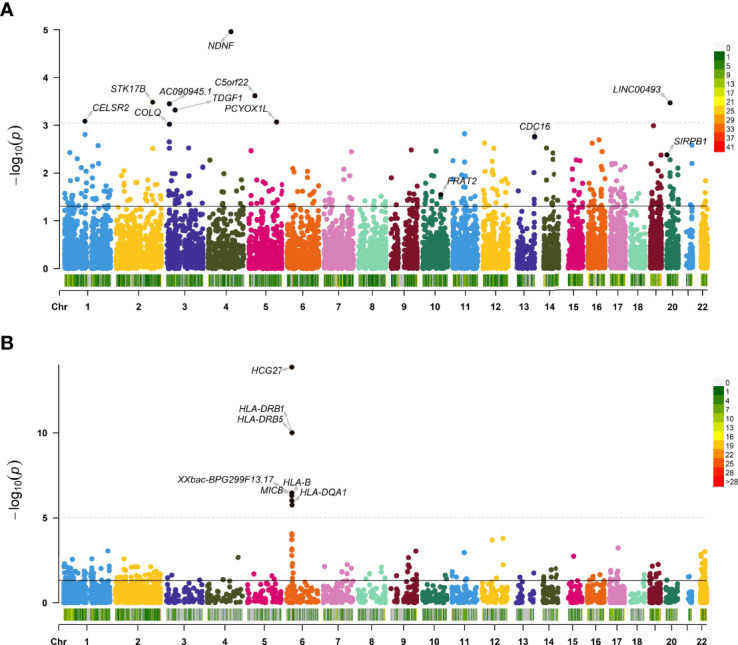
Manhattan plot showing transcriptome-wide association study (TWAS)–identified genes. Manhattan plot showing TWAS-identified genes and significantly expressed genes associated with juvenile idiopathic arthritis (JIA; annotated points) for Asian **(A)** and European **(B)** populations. Each point represents a single gene, and the physical position (chromosome localization) is plotted on the x-axis, whereas the −log_10_ (*P*-value) of the association between gene and JIA is plotted on the y-axis. TWAS, transcriptome-wide association study; JIA, juvenile idiopathic arthritis.

For Asian population, 350 significant genes were found (**Figure 1A**). Among them, there were 293 genes with *P* < 0.05 for skeletal muscle and 80 for whole blood (23 genes expressed in both tissues), including *NDNF* (*P* = 1.10E-05), *C5orf22* (*P* = 2.41E-04), and *STK17B* (*P* = 3.30E-04) (**Supplementary Table 1**).

### Transcriptome-Wide Association Study Analysis Results of the European Population

For European, TWAS identified 195 significant genes using these data (**Figure 1** **B**). A total of 176 genes with *P* < 0.05 were found for skeletal muscle, and 127 were found for whole blood (108 genes expressed in both tissues), such as *HCG27* (*P* =1.36E-14), and *HLA-DRB1* (*P* = 9.80E-11) (**Supplementary Table 2**). The 10 top significant genes in the Asian and European group are shown in **Table 1**, respectively, including, population, heritability of genes (HSQ), the BEST.GWAS.ID, number of SNPs in the locus (NSNP), and TWAS *P*-value (P TWAS).

**Table 1:** Top genes selected by transcriptome-wide association study (TWAS) analysis (Asian and European).

Population	Gene	CHR	BEST.GWAS.ID	NSNP	TWAS.Z	TWAS.P
Asian	NDNF	4	rs13128284	472	4.3960	1.10E-05
	C5orf22	5	rs584128	675	3.6716	2.41E-04
	STK17B	2	rs3736516	376	-3.5904	3.30E-04
	LINC00493	20	rs6035106	474	3.5811	3.42E-04
	AC090945.1	3	rs2455833	474	-3.5710	3.56E-04
	TDGF1	3	rs3806702	374	-3.4914	4.80E-04
	CELSR2	1	rs4970834	423	-3.3446	8.24E-04
	PCYOX1L	5	rs1833661	568	-3.3346	8.54E-04
	COLQ	3	rs2455833	383	-3.3052	9.49E-04
	CTC-429P9.5	19	rs6512158	380	3.2810	1.03E-03
European	HCG27	6	rs3819299	168	-7.6997	1.36E-14
	HLA-DRB5	6	rs28421666	248	6.4700	9.80E-11
	HLA-DRB1	6	rs28421666	233	6.4700	9.80E-11
	XXbac-BPG299F13.17	6	rs3819299	168	5.0986	3.42E-07
	MICB	6	rs3819299	264	-5.0312	4.87E-07
	HLA-B	6	rs3819299	207	4.9008	9.54E-07
	HLA-DQA1	6	rs28421666	210	4.7800	1.75E-06
	XXbac-BPG248L24.12	6	rs3819299	207	3.9294	8.52E-05
	HLA-DQB1	6	rs28421666	217	3.8859	1.02E-04
	RP3-462E2.3	12	rs653178	203	3.7730	1.61E-04

The large-scale Genome-Wide Association Study (GWAS) summary data for juvenile idiopathic arthritis (JIA) acquired from Asian and European cohort studies, including 898 JIA patients and 346,102 controls. The TWAS.P and TWAS.Z values were calculated by the FUSION approach (http://gusevlab.org/projects/fusion/).

TWAS, transcriptome-wide association study; GWAS, Genome-Wide Association Study; JIA, juvenile idiopathic arthritis; TWAS P, TWAS P-value; TWAS Z, TWAS Z-score; NSNP, number of SNPs in the locus.

### Common Significant Genes Identified by Transcriptome-Wide Association Study for Asian and European Populations

**Figure 2:**
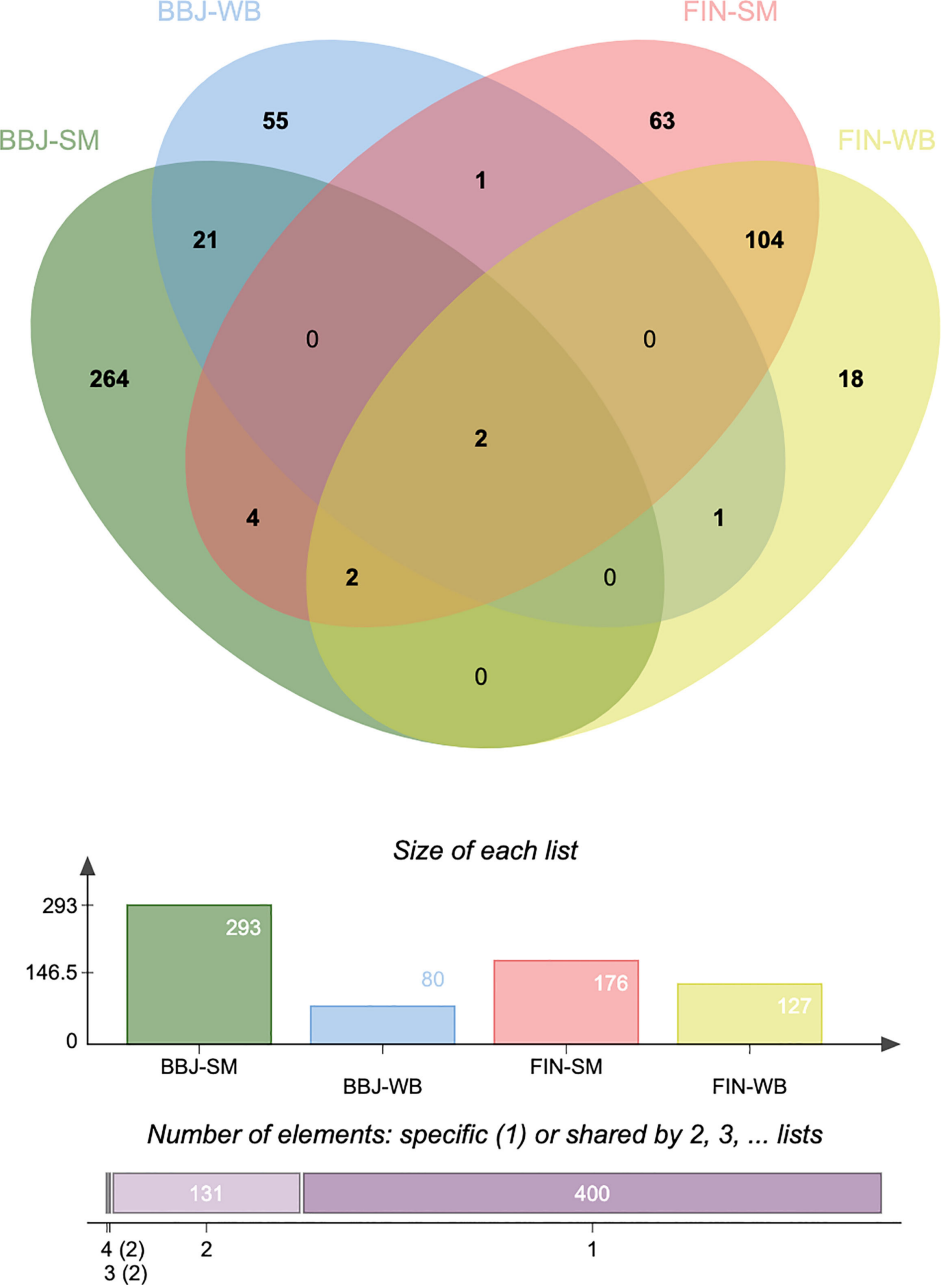
Venn diagram reveals the overlap of transcriptome-wide association study (TWAS)-significant genes in different populations and tissues. TWAS: transcriptome-wide association study; BBJ, BioBank Japan; FIN, FinnGen; SM, skeletal muscle; WB, whole blood.

To find out the most overlapped and representative genes, we performed an overlap analysis of genes in different tissues and populations. The Venn diagram (**Figure 2**) shows the number of genes expressed in one or more tissues/populations. A total of 10 genes are expressed in both the Asian and European populations, such as *CDC16* (*P* = 0.00172) and *PSMD5-AS1* (*P* = 0.0276) (**Supplementary Table 3**). It is noticeable that half of these overlapped genes were expressed in both two tissues. These genetic loci shared by different populations and tissues of JIA nominated candidate mechanisms underlying JIA. It is also noticed that several of the most significant genes were located on chromosome 6 and that the rsID of the most significant GWAS SNP in the locus (BEST.GWAS.ID) was SNP rs3819299. If this allele mutated, it will be altering the expression of downstream target genes.

### Validating Transcriptome-Wide Association Study Results With the mRNA Expression Profiles of Juvenile Idiopathic Arthritis

**Figure 3:**
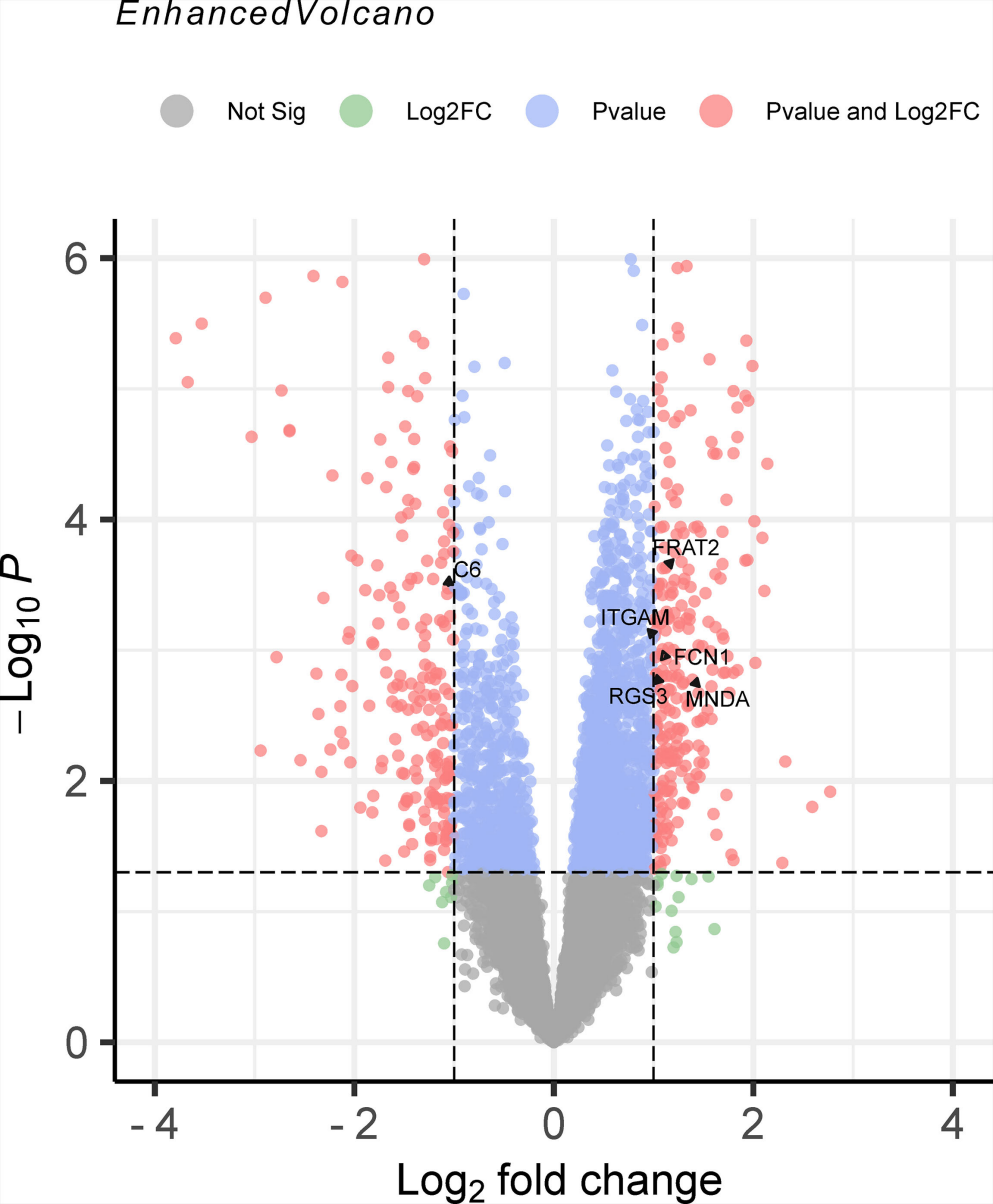
The volcano plot of mRNA expression profiles for juvenile idiopathic arthritis (JIA). The results of mRNA expression profiles were output to the volcano map. Genes were marked in red point as differentially expressed when the following two conditions were met: *P* < 0.05 by the moderated *t* statistic and |logFC| > 1. JIA, juvenile idiopathic arthritis; logFC, log fold change.

Comparing the TWAS results with the mRNA expression profiles results, eight common genes were identified and showed in **Table 2**, including *SIRPB1* (*P*_TWAS_ = 4.21E-03, *P*_DEG_ = 1.50E-04), and *FRAT2* (*P*_TWAS_ = 2.82E-02, *P*_DEG_ = 1.43E-02). The distribution of gene expression was visualized in the corresponding volcano plot (**Figure 3**).

**Table 2:** The common genes identified by both transcriptome-wide association study (TWAS) and differentially expressed genes (DEGs) for juvenile idiopathic arthritis (JIA).

Tissue	Gene	Chromosome	*P*_TWAS_	*P*_DEG_	Regulation
SM+WB	FCN1	9	8.90E-04	2.73E-02	UP
SM	SIRPB1	20	4.21E-03	1.50E-04	UP
SM	C6	5	1.40E-02	1.45E-02	Down
WB	FRAT2	10	2.82E-02	1.43E-02	Up
WB	ITGAM	16	3.85E-02	2.24E-02	Up
WB	RGS3	9	3.77E-02	3.30E-02	Up
WB	MNDA	1	3.27E-02	3.40E-02	UP

Each P_TWAS_ value was calculated by transcriptome-wide association study (TWAS) analysis. Each P_DEG_ value was the differentially expressed gene (DEG) derived from the published studies.

TWAS, transcriptome-wide association study; DEG, differentially expressed gene; JIA, juvenile idiopathic arthritis; P_TWAS_, P transcriptome-wide association study value; P_DEG_, P differentially expressed gene value; SM, skeletal muscle; WB, whole blood.

### Functional Examination of the Transcriptome-Wide Association Study–Identified Genes Associated With Juvenile Idiopathic Arthritis

**Figure 4:**
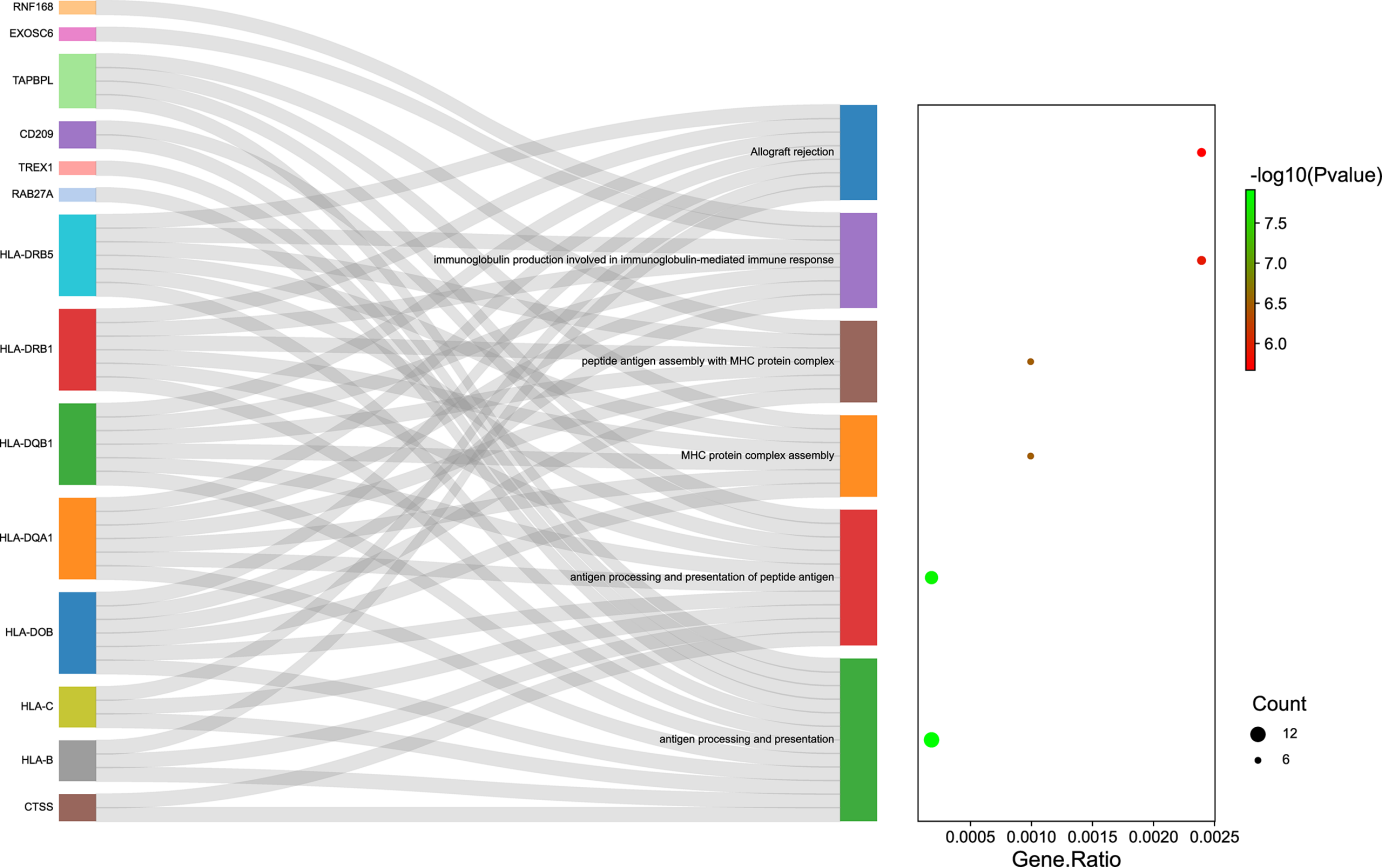
The top overrepresented Gene Ontology (GO) terms and related genes. Sankey plot showed the relationship between the genes and overrepresented GO terms. The dot plot showed the ratio between the genes identified involved in GO terms and the total number of genes included in each GO terms (FDR *P* ≤ 0.05). GO, Gene Ontology.

In this study, pathway and process enrichment analysis was carried out with the following ontology sources: GO biological processes, KEGG Pathways, GO molecular functions, reactome gene sets, canonical pathways, and Resource for Mammalian Protein Complex (CORUM). The 535 genes identified by TWAS analysis in Asian and European populations were all successfully submitted to Metascape to perform GO enrichment analysis. Metascape identified 183 terms for the TWAS results, including 114 GO terms such as antigen processing and presentation (GO:0019882) and 25 KEGG pathways such as phagosome (hsa04145) (**Supplementary Table 4**). The significant terms were then hierarchically clustered, selected as a subset of representative terms, and converted into a network layout (**Supplementary Figure 1**). **Supplementary Figure 1**, **Supplementary Table 4** show that the most significant biological pathways were antigen processing and presentation, adaptive immune system, and neutrophil degranulation. The Sankey and dot plots show the top overrepresented GO terms and related genes belonging to the result of enrichment analysis results (**Figure 4**). By comparing the Metascape results of TWAS analysis and mRNA expression profiles, 19 terms were identified, such as rheumatoid arthritis (hsa05323) and cytokine signaling in immune system (R-HSA-1280215) (**Supplementary Table 5**).

## Discussion

JIA is a heterogeneous inflammatory rheumatic condition with different incidence and prevalence between populations (20, 27). Previous studies have identified 22 genome-wide significant loci associated with JIA, but the ability to interpret the relationship between the significant genes and JIA is limited (28, 29). To reduce millions of GWAS generated non-sense results and determine more potential genetic mechanisms in genetic aetiology, we conducted a TWAS analysis by using large-scale GWAS summary datasets acquired from Asian and European cohorts. This also could eliminate ethnicity factors between different populations.

By comparing the TWAS results of different tissues and populations, we identified some significant genes, such as *CDC16*. The *CDC16*, also called *APC6*, is a component protein of anaphase promoting complex/cyclosome (APC/C) complex, involving in the process of chromosome segregation during mitosis (30). This protein participates several pathways including cellular response to stimuli, cellular responses to stress, and cellular senescence, which may response to hypoxia, starvation, heat stress, and cellular senescence. Part of the factors also played important roles in other arthritis (31, 32). The *TBC (Tre-2/Bub2/Cdc16)* domain was originally identified as a conserved domain, and it is now widely recognized as a conserved protein motif that consists of approximately 200 amino acids in almost all eukaryotes (33). In a recent study, *TBC1* domain family member 3 *(TBCID3)* was shown to regulate the cargo and biological activity of extracellular vesicles (EVs), mediating tissue repair. These EVs represent a ubiquitous mechanism of cell–cell communication that is critically important for inflammation, immune tolerance, and arthritis (34–37).

By integrating the TWAS analysis and mRNA expression profiles of JIA, we identified several common genes, such as *FCN1*, *HLA-RB5*, *SIRPB1*, and *FRAT2. FCN1* was the only one gene upregulated in both whole blood and skeletal muscle of JIA patients. Polymorphisms in the ficolin 1 gene *(FCN1)* are associated with susceptibility to the development of rheumatoid arthritis (RA) (38). It is associated with RA and participates innate immune system that is persistently activated in RA (39). The *HLA-RB5* gene is in the HLA region of chromosome 6p21.31, and various studies have identified the HLA gene region as a major susceptibility locus for JIA, including HLA class I (*HLA A-2, and HLA B27)* and HLA class II (*HLA-DRB1 and HLA DP*) genes (40, 41). Some of the members are associated specific subtype of JIA and adult arthritis. HLA-DRB1*04 is associated with Rheumathoid Factor (RF)-positive polyarthritis in children and RF-positive RA in adults, whereas HLA-DRB1*08, HLA-DRB1*11, and HLA-DRB1*13 are strongest associated with RF-negative polyarthritis and oligoarticular JIA (42, 43). HLA is primarily involved in immune function. Previous studies have also shown that activated T cells accumulate in the synovium and can trigger an autoimmune response in JIA. These findings illustrate the important role of T cells in the disease course (44, 45). Recently, a study showed that *HLA-RB5* plays a role in the expansion of T lymphocytes in primary Sjogren’s syndrome, which indicates that *HLA-RB5* may similarly impact autoimmune diseases such as JIA (46).

*SIRPB1* belongs to the signal-regulatory protein (SIRP) family of genes, which encode cell-membrane proteins that are mainly expressed on myeloid and neural cells. It interacts with *TYROBP/DAP12*, which actives immune cells to trigger an inflammatory response to injury or disease (47). Studies show that the expression of *DAP12* is increased in RA patients, and blocking *DAP12* with a tumor necrosis factor (TNF) inhibitor could reduce the signaling and cytokine production (48, 49). In addition, previous studies have shown that DAP12 has an important role in bone metabolism, and the *DAP12*-mediated recruitment of inflammatory macrophages and neutrophils to the joint and promotion of bone erosion are key factors in autoimmune joint diseases (50). *SIRPB1* also participates in the recruitment of spleen tyrosine kinase (SYK) that mediates several key functions including innates immune recognition, osteoclast maturation, platelet activation, and vascular development (51). Blocking of SYK could inhibit the immune complex-mediated inflammation in arthritis (52). Based on all above, *SIRPB1* showed strong association with autoimmune diseases, which may be involved in the mechanisms of JIA and provide opportunities to develop new drugs that act on these targets.

*FRAT2* is a Wnt signaling pathway regulator on human chromosome 10q24.1 (53). It has been reported that the *FRAT2* upregulation is related to the activation of the Wnt signaling pathway (54, 55). WNT signaling plays an essential role in embryonic development and tissue homeostasis in adults; thus, abnormal regulation of Wnt signaling is linked to a variety of musculoskeletal diseases, such as osteoporosis (56). Previous studies have shown Wnt signaling functions in the regulation of cartilage development, growth, and maintenance, influencing skeletal diseases, including arthritis and JIA (57, 58). Studies have shown that Wnt pathway inhibitors reduces the progression of osteoarthritis (59, 60).

GO enrichment analysis identified several biological pathways associated with JIA. Most of these pathways are related to the immune system, such as the regulation of leukocyte activation, positive regulation of T-cell activation, cytokine signaling in the immune system, and cell adhesion molecules. JIA is an immune system disease. The autoreactive immune response in JIA is thought to be initially triggered by an adaptive response to a self-antigen, and soon after the initial autoreactive insult, almost all of the immune system takes part in the immune response (41). Cell adhesion molecules (hsa04514) are glycoproteins expressed on the cell surface that play important roles in inflammatory diseases; the activation, migration, and penetration of leucocytes into local inflammatory tissues are dependent on attachment to adhesion molecules on endothelial cells (61). Chronic tissue inflammation and damage caused by leucocytes are pathological markers of JIA, and some studies have shown that the levels of soluble E-selectin (sE-selectin) and soluble intercellular adhesion molecule-1 (*sICAM-1)* in JIA patients are significantly higher than those in normal controls in both the active stage and clinical remission (62, 63). These studies demonstrated the role of cell adhesion molecules in the pathogenesis of JIA.

Additionally, the positive regulation of T-cell activation (GO:0050870) was identified as a pathway associated with JIA. Research has demonstrated a key role for aberrant T-cell activation pathways in the initiation and perpetuation of arthritis (64). In JIA, activated memory T cells accumulates in the synovium, and an elevated level of T helper 17 (T_H_17) cells accompanied by a decrease in the regulatory T-cell (T_reg_) population is observed in JIA patients (41, 65). Overall, these identified pathways may deepen understanding of the immune effects of JIA and provide new directions for future investigations.

It is noticed that several of the most significant genes were located on chromosome 6 and BEST.GWAS.ID was SNP rs3819299. A previous study found several MHC Loci such as HLA-DRB1*11, which located in chromosome 6 influences JIA susceptibility (43). What’s more, in JIA patients, the expression of complement located in chromosome 6 such as *C4* located was significantly reduced compared with controls (66). These existing researches were consistent with our results.

In this study, overlap analysis of the TWAS results in the two populations was performed, and we understand the shared genetic underpinnings of JIA patients with different ethnicities, which addressed the potential effects on JIA introduced by the confluence of race and ethnicity. In addition, this study underscored the presence of differences in JIA across different racial/ethnic groups. In other words, this study preliminarily found the heterogeneity of JIA. There are some possible mechanisms for the gene differences between Asian and European. First, several epidemiologic surveys documented a marked, disparity in the prevalence of JIA among different racial/ethnic groups (67–69). Second, the subtypes distribution of JIA may be significant among the different ethnic groups. The specific mechanisms for the gene differences in JIA needed further research.

We explored the genetic mechanisms of JIA in different populations by using TWAS analysis. As an innovative method, TWAS analysis can predict gene expression in JIA in the absence of confounding factors from environmental diversity that may influence gene expression. In addition, analyzing multiple populations can increase the validity of the results. To further validate the TWAS results, we verified the candidate genes by comparing them with the mRNA expression profiles. However, there are some limitations. First, one of the JIA GWAS data was derived for Asian populations, but the TWAS reference weights were based on European populations. Further TWAS reference weights on other populations are needed to prove our results. Second, we found some candidate genes are located on major histocompatibility complex (MHC) locus in the chromosome 6. However, the genetic variation in the MHC locus is so complicated that the genes (such HLA genes) and molecular mechanisms accounting for this should be cautioned to use.

## Conclusions

In summary, by using GWAS summary datasets from Asia and Europe, TWAS analysis identified novel and common susceptibility genes for JIA. Our results provide novel clues for understanding the genetic mechanism of JIA, focusing on the possible roles of candidate genes in the pathogenesis of JIA. In addition to specific mechanistic findings for JIA, this work also outlines a systematic approach for identifying functional mediators of complex diseases.

## Data Availability Statement

The datasets presented in this study can be found in online repositories. The names of the repository/repositories and accession number(s) can be found in the article/**Supplementary Material**.

## Author Contributions

(I) Conception and design: JX, BS. (II) Administrative support: JM, HS, BS. (III) Provision of study materials: JX, YZ, HS. (IV) Collection and assembly of data: JX, HS. (V) Data analysis and interpretation: JX, YW, SZ. (VI) Manuscript writing: JX JM. (VII) Final approval of manuscript: All authors

## Funding

This work was supported by the National Natural Science Foundation of China (grant number 81974347 and 81802210); the Department of Science and Technology of Sichuan Province (grant number 2021YFS0122). Financial support had no impact on the outcomes of this study.

## Conflict of Interest

The authors declare that the research was conducted in the absence of any commercial or financial relationships that could be construed as a potential conflict of interest.

## Publisher’s Note

All claims expressed in this article are solely those of the authors and do not necessarily represent those of their affiliated organizations, or those of the publisher, the editors and the reviewers. Any product that may be evaluated in this article, or claim that may be made by its manufacturer, is not guaranteed or endorsed by the publisher.

## Supplementary Material

Supplementary Figure 1 The network layout of representative Gene Ontology (GO) terms. The network layout of representative GO terms under hierarchical clustering. In the network, each circle node represents a term, where its size is proportional to the number of input genes fall into that term, and its color represents its cluster identity (i.e., nodes of the same color belong to the same cluster). Terms with a similarity score > 0.3 are linked by an edge (the thickness of the edge represents the similarity score). GO, Gene Ontology.

## Abbreviations

BBJ, BioBank Japan; eQTL, Expression Quantitative Trait Loci; EVs, extracellular vesicles; FCN1, Polymorphisms in the ficolin 1; FUSION, Functional Summary-Based Imputation; GEO, Gene Expression Omnibus; GO, Gene Ontology; GWAS, Genome-wide association study; JIA, juvenile idiopathic arthritis; KEGG, Kyoto Encyclopedia of Genes and Genomes; LD, linkage disequilibrium; LogFC, Log fold change; RA, rheumatoid arthritis; sICAM-1, soluble intercellular adhesion molecule-1; SIRP, signal-regulatory protein; SYK, spleen tyrosine kinase; T_H_17, T helper 17; TWAS, Transcriptome-wide association study.
