# Supplementary figures and images for: A Cross-Tissue Transcriptome-Wide Association Study Identifies Novel Susceptibility Genes for Juvenile Idiopathic Arthritis in Asia and Europe
Source: Front Immunol. 2022 Jul 28;13:941398. doi: 10.3389/fimmu.2022.941398 (PMC9367689; doi:10.3389/fimmu.2022.941398)

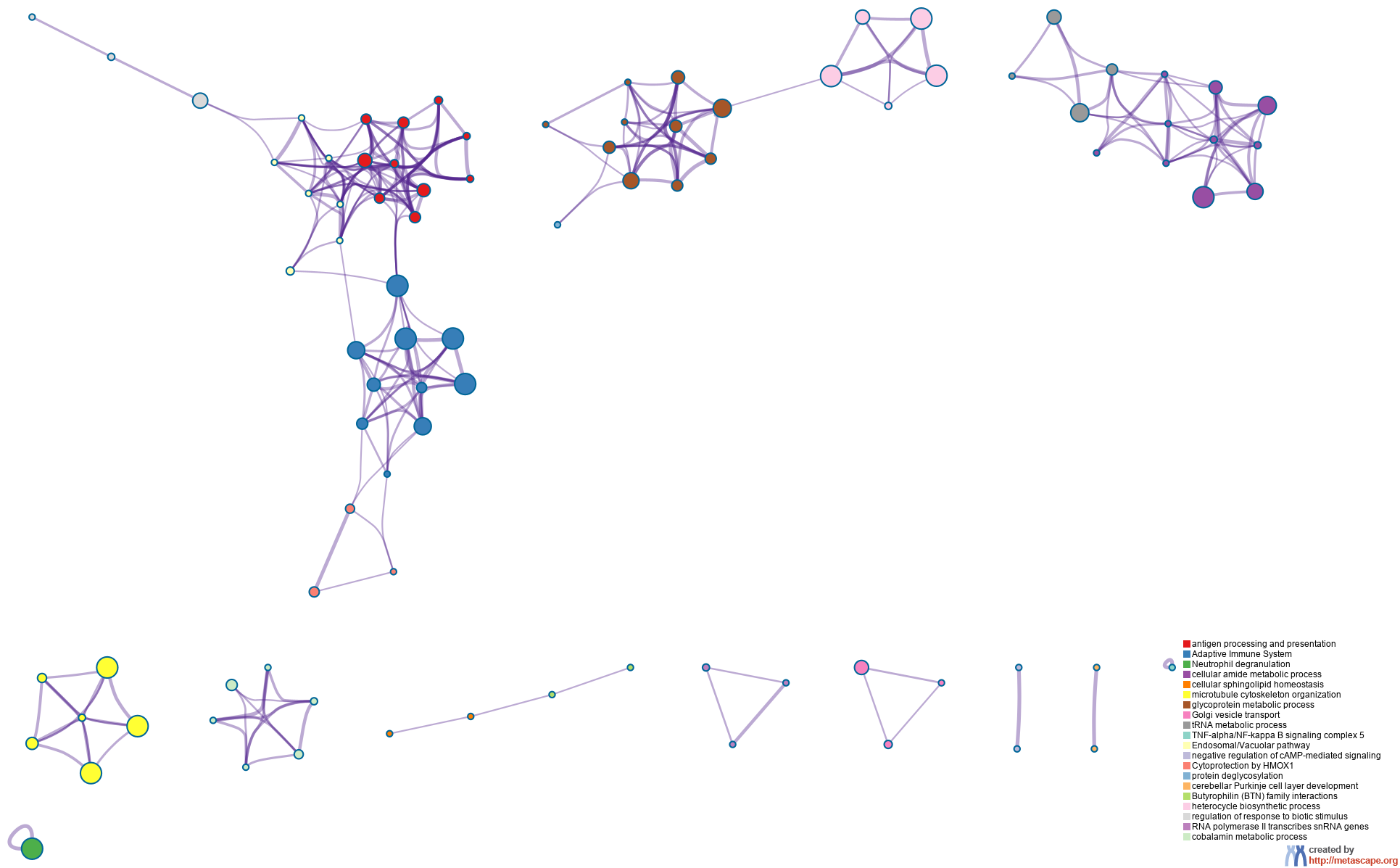

Supplement: Supplementary Figure 1 — The network layout of representative Gene Ontology (GO) terms. The network layout of representative GO terms under hierarchical clustering. In the network, each circle node represents a term, where its size is proportional to the number of input genes fall into that term, and its color represents its cluster identity (i.e., nodes of the same color belong to the same cluster). Terms with a similarity score > 0.3 are linked by an edge (the thickness of the edge represents the similarity score). GO, Gene Ontology. [file Image_1.jpeg]
